# Paired-omics-based exploration and characterization of biosynthetic diversity in lichenized fungi

**DOI:** 10.1099/mgen.0.001569

**Published:** 2025-12-17

**Authors:** Garima Singh, Maonian Xu, Mitja Zdouc, Anna Pasinato, Susan Egbert, Xinhui Yu, Elin Soffia Olafsdottir, Nuria Beltran-Sanz, Pradeep K. Divakar, David Pizarro, Jordan R. Hoffman, Christoph Scheidegger, Imke Schmitt, Francesco Dal Grande, Marnix H. Medema

**Affiliations:** 1 Department of Biology, University of Padova, Via U. Bassi, 58/B, 35121 Padova, Italy; 2 Botanical Garden, University of Padova, Padua, Italy; 3 National Biodiversity Future Center (NBFC), Piazza Marina, 61, Palermo, 90133, Italy; 4 Faculty of Pharmaceutical Sciences, University of Iceland, Hagi, Hofsvallagata 53, IS-107 Reykjavik, Iceland; 5 Bioinformatics Group, Wageningen University, Droevendaalsesteeg 1, 6708PB Wageningen, The Netherlands; 6 University of Manitoba, 66 Chancellors Cir, Winnipeg, MB R3T 2N2, Canada; 7 Department of Pharmaceutical Sciences, College of Pharmacy, Oregon State University, Corvallis, Oregon, 97331, USA; 8 Department of Pharmacology, Pharmacognosy and Botany, Faculty of Pharmacy, ComplutenseUniversity of Madrid(UCM), Madrid28040, Spain; 9 Science and Mathematics Division, Delta College, 1961 Delta Rd., University Center, MI, USA; 10 Biodiversity and Conservation Biology, Swiss Federal Institute for Forest, Snow and Landscape Research, WSL, Zürcherstr. 111, CH-8903 Birmensdorf, Switzerland; 11 Senckenberg Biodiversity and Climate Research Centre (SBiK-F), Frankfurt Am Main, 60325, Germany; 12 Department of Biosciences, Institute of Ecology Evolution and Diversity, Goethe University Frankfurt, Max-Von-Laue-Str. 13, Frankfurt am Main, 60438, Germany

**Keywords:** alectoronic acid BGC, antiSMASH, biosynthetic genes, BGCs, BiG-SCAPE, depsides, depsidones, evernic acid BGC, GCFs, lichens, natural products, perlatolic acid BGC, secondary metabolism, stenosporic acid BGC

## Abstract

The increasing demand for novel drug leads requires bioprospecting non-model taxa. Comparative genomics and correlative omics are a fast and efficient method for linking bioactive but genetically orphan natural products to their biosynthetic gene clusters (BGCs) and identifying potentially novel drug leads. Here we implement these approaches for the first systematic comparison of the BGC diversity in lichen-forming fungi (LFF) (comprising 20% of known fungi), prolific but underutilized producers of bioactive natural products. We first identified BGCs from all publicly available LFF genomes (111), encompassing 71 fungal genera and 23 families, and generated BGC similarity networks of each class. We recovered 5,541 BGCs grouped into 4,464 gene cluster families. We used mass spectrometry (MS) and correlative metabolomics to link five MS-identified metabolites – alectoronic acid, alpha-collatolic acid, evernic acid, stenosporic acid and perlatolic acid – to their putative BGCs. We subsequently used MS on an additional 80 species to explore the taxonomic breadth of common lichen compounds, uncovering a strong pattern between specific families and secondary metabolites. We found that (1) ~98% of the BGCs in LFF are putatively novel (uncharacterized to date), (2) lichen metabolic profiles contain a plethora of unidentified metabolites and (3) ribosomal peptide-related BGCs constitute about 20% of the LFF BGC landscape. Our study provides comprehensive insights into the BGC landscape of LFFs, highlighting unique, widespread and previously uncharacterized BGCs. We anticipate that the approach we describe will serve as a baseline for leveraging biosynthetic research in non-model organisms, inspiring further investigations into microbial dark matter.

**Impact Statement**

This study presents the first large-scale, systematic analysis of biosynthetic gene cluster (BGC) diversity in lichen-forming fungi (LFF), a group that represents nearly 20% of known fungal diversity but remains largely unexplored in terms of biosynthetic potential and the diversity of various biosynthetic gene classes. By analysing 111 genomes across 71 genera and 23 families, we reveal that ~98% of LFF BGCs are putatively novel, with a surprisingly high prevalence of ribosomally synthesized peptide clusters. Furthermore, through integrative metabolomics, genome mining and gene network clustering, we link five key lichen bioactive metabolites to their biosynthetic pathways and uncover strong taxonomic-family-level patterns in metabolite production. Linking metabolites to their BGC is essential for enabling heterologous expression and the controlled production necessary for future therapeutic applications. Our findings shed light on the immense untapped biosynthetic capacity of LFF and establish a critical foundation for future research into natural product discovery from non-model fungi.

## Data Summary

Supplementary information

**S1** Sample voucher information along with the taxonomic information and total number of BGCs and the numbers of PKS, NRPS, RiPP and terpene BGCs.

**S2** Overview of biosynthetic genes by BGC class and taxa

**S3** (A) Number of gene cluster families (GCFs) for all the BGC classes and per taxon, as identified by BiG-SCAPE. (B) Number of GCFs per taxon identified by the BiG-SCAPE to demonstrate the sources of most unique BGCs in lichens

**S4** BGCs identified based on clustering with atranorin, usnic acid and gyrophoric acid gene clusters.

**S5** GenBank files of the BGCs deorphanized in this study: https://doi.org/10.5281/zenodo.15521901

**S6** Voucher information of the taxa used for the MS

## Introduction

Natural products (NPs) are a crucial source of bioactive compounds. The majority of modern medicines and therapeutic agents, including most anticancer compounds and antibiotics, are derived from plant and microbial NPs [1,2]. However, the emergence of novel infections combined with the appearance of antibiotic-resistant pathogens is posing an increasing demand for novel drugs to combat the global health crises [3,4]. The emergence of viral epidemics and drug resistance also points toward the urgent need for novel antimicrobial therapy [3,6]. Researchers have begun to explore novel organisms, as well as classes of biosynthetic genes, for their pharmaceutically usable bioactive properties. This includes biosynthetic gene clusters (BGCs) as ribosomally synthesized and post-translationally modified peptides (RiPPs), owing to their strong antimicrobial activities and high stability, specificity and affinity for targets, cellular penetrability and great engineering potential [1,7,10].

 Advances in genome sequencing, biosynthetic gene discovery pipelines and synthetic biology approaches enable the utilization of accumulating genomic data for culture-free bioprospecting of non-model organisms, addressing key bottlenecks in natural product discovery [11,12]. For instance, while lichenized fungi – fungi living in obligate symbiotic association with one or more photobionts and other associated microbiota [13,17] – are a treasure trove of secondary metabolites [18,19], with around 1,000 described to date and many exhibiting strong bioactivity, the industrial applications of lichen-derived compounds remain underutilized. This is primarily attributed to the experimental challenges inherent in symbiotic systems, the slow growth rate of lichens, difficulties in generating axenic mycobiont culture biomass and the lack of known triggers to stimulate metabolite synthesis [20]. While lichen genomes contain genes for synthesizing a diverse array of metabolites – non-ribosomal peptide synthetases (NRPSs), polyketide synthases (PKSs), hybrid NRPS–PKSs, terpene synthases and RiPP synthases – the biosynthetic research on lichenized fungi has primarily focused on PKSs [11,17, 21,25] and more recently on terpene-related BGCs [26]. In fact, genome mining efforts have revealed a substantial gap between the total BGCs present in genomes and those that have been experimentally/bioinformatically characterized [20,27, 28]. This suggests the existence of a large number of uncharacterized or unlinked BGCs, often referred to as ‘silent’ or ‘orphan’ BGCs. One reason for the focus on PKSs is because most of the well-known, commonly analysed lichen compounds are PKS-derived (e.g. depsides, depsidones, xanthones, anthraquinones and dibenzofurans [22,24, 28, 29]). Computational approaches can bypass culture dependency and expand NP exploration beyond PKSs to enable the identification, dereplication and prioritization of the entire biosynthetic diversity of organisms.

 The genes encoding NPs often lie adjacent to each other in a linear fashion, forming BGCs [30,32]. A BGC typically has one or a few of the following core genes that define the chemical class of the encoded compound, such as NRPSs, PKSs, terpene synthases or RiPP synthases [32,33]. Recently, automated pipelines have been developed to facilitate the bioinformatic linking of genes to molecules through similarity-based clustering of homologous BGCs into gene cluster families (e.g. BiG-SCAPE [34] and BiG-SLiCE [35]). This approach not only facilitates the linking of BGCs to their putative metabolites but also enables the prediction of biosynthetic functions and novelty by comparing them with characterized BGCs in databases such as MIBiG [36]. Studies implementing BGC clustering approaches on bacteria reveal that we have explored only a tiny fraction of the available diversity for its biosynthetic potential [37,39]. In lichenized fungi, only a few BGCs have been linked to the metabolites (e.g. grayanic acid [24], atranorin [11], lecanoric acid [12], usnic acid [22,28, 40] and olivetoric acid [41]), and metabolic diversity is known only for the commonly analysed compound classes, e.g. those listed in [42,43]. A comprehensive analysis of the total metabolic potential of lichen-forming fungi (LFF) is still lacking. With the recent surge in genomic data and the development of advanced BGC prediction and clustering pipelines, there is now an opportunity to better understand the BGC diversity and novelty in lichenized fungi, enabling informed predictions about the most industrially relevant taxa and metabolites.

 Here, we investigate the metabolic diversity in lichenized fungi using a comparative genomics and molecular networking approach to bioinformatically characterize the lichen ‘BGC landscape’. We further employ correlative omics to link five bioactive metabolites to their biosynthetic origins. In addition, we assessed the taxonomic distribution of the most prevalent lichen metabolites by performing MS on 80 LFF. Specifically, we aim to (1) unravel the extent of biosynthetic novelty encoded in lichen-forming fungal genomes, (2) deorphanize common and potent bioactive metabolites using comparative metabolomics, network and clustering approaches and (3) estimate the taxonomic breadth of common lichen metabolites. We anticipate that the high percentage of novel BGCs in non-model organisms such as lichens, as highlighted in this study, will inspire investigations into gene functions and metabolic gene clusters of underexplored taxa.

## Methods

### Dataset, genome assembly and annotation

**Fig. 1.**
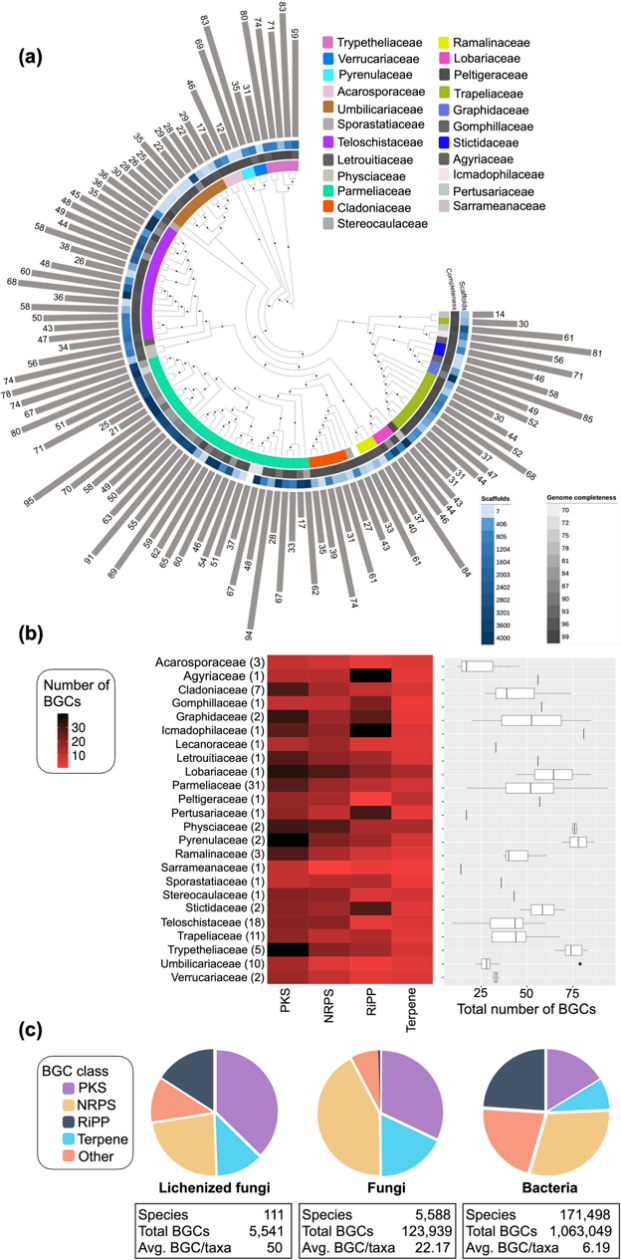
Genome quality metrics and diversity of biosynthetic genes in lichenized fungi. (a) Phylogenomic tree displaying the genome quality (genome completeness and number of scafffolds) and number of BGCs. (**b**) BGC diversity among families of *Lecanoromycetes*. The heat map and box plot illustrate the distribution of BGCs across taxonomic families in *Ascomycota*. The heat map represents the relative abundance of each BGC class, with red indicating lower values and black indicating higher values. These values are calculated as the total number of each BGC class divided by the number of taxa sampled within each family. The box plot shows the range of total BGC counts per family, with the mean indicated by the line within each box. A narrow, vertical line in the box plot suggests that the corresponding family includes only a single species. (**c**) BGC diversity in lichenized fungi compared to fungi and bacteria. The biosynthetic landscapes of lichenized fungi, non-lichenized fungi and bacteria differ markedly, with the most striking contrasts observed in RiPPs and NRPSs between lichenized and non-lichenized fungi, and in relative number of PKSs present in bacteria and lichenized fungi. The exact values are listed in Material S2; source [73].

A total of 111 taxa were included in the study (Material S1, available in the online Supplementary Material), from 71 genera from 23 lichenized fungal families (Fig. 1a), encompassing broad phylogeographic ranges and ecological niches. This dataset includes all *Lecanoromycetes* genomes available up to January 2024 (151 in total), on which the following filters were applied: (1) genome completeness greater than 95%, (2) retention of a single representative genome per species, (3) assemblies containing fewer than 10,000 scaffolds, and (4) inclusion of genomes of LFF from *Eurotiomycetes* and *Dothideomycetes* (9 in total). Recent studies using similar datasets [26], comprising all available LFF genomes along with selected non-lichenized fungal genomes, have focused on identifying conserved terpene BGCs across lichens. In this study, we aim to investigate the distribution of biosynthetic diversity across different LFF families, with the goals of linking metabolites to specific BGCs and assessing the extent of potentially unique or novel BGCs in lichens.

For the functional annotation of genomes, including genes and proteins, prediction was performed with scripts implemented in the funannotate pipeline [44]. The genomes were first masked for repetitive elements, followed by gene prediction using BUSCO2 to train Augustus and self-training GeneMark-ES [45]. The functional annotation of the predicted genes was performed with InterProScan (51), egg-NOG-mapper [46] and BUSCO [47,48] using Ascomycota_db models. Secreted proteins were predicted using SignalP as implemented in funannotate (43) ‘annotate’ command.

### Genome completeness assessment and phylogenomic analysis

We used the BUSCO Ascomycota dataset to estimate genome completeness [47,48]. The single-copy BUSCOs from 111 taxa were quality-filtered and then compared to filter out those present in most taxa (a maximum of one sample missing). BUSCO genes that passed the above steps were then selected for generating the phylogenomic tree. For each taxon, the single-copy BUSCOs were concatenated, and the concatenated sequences from all the taxa were then aligned using MAFFT l-INS-i. Evolutionary relationships were inferred from this multiple sequence alignment using maximum likelihood (ML) analysis implemented in IQ-TREE v1.5.5 [49,50] with standard model selection and 1,000 bootstrap replicates. The resulting tree was visualized using FigTree 1.3.1 [51] and annotated in iTOL [52].

### BGC prediction and clustering

BGCs were predicted and annotated using antiSMASH (antibiotics and SM Analysis Shell, v7.0 [53]) (Material S2). To quantify BGC diversity, we used Biosynthetic Genes Similarity Clustering and Prospecting Engine (BiG-SCAPE [34]) (https://git.wageningenur.nl/medema-group/BiG-SCAPE), a platform for comparing and grouping similar BGCs into gene cluster families (GCFs) based on distance matrices. BGCs assigned to a GCF potentially encode structurally similar natural products. BGCs that do not group with a MIBiG reference BGC code for putatively novel natural products. AntiSMASH results were compared against the MIBiG database of characterized BGCs using BiG-SCAPE. We computed the BGC assignment into GCF using the raw distance cut-offs of 0.20, 0.4, 0.6 and 0.80. The lower the cut-off is, the stricter the similarity clustering, resulting in fewer connections. We used a conservative approach and a cut-off of 0.6 to avoid overestimating the number of potentially novel BGCs. All the analyses (i.e. with different thresholds) were performed using the default settings with the ‘auto’ mode, with singletons retained and with the PFAM database.

### Identification of BGCs with known and unknown compounds and potentially novel BGCs

Novel BGCs comprise both BGCs with yet-unknown chemical products, as well as BGCs encoding the biosynthesis of known products, for which the biosynthesis has not yet been elucidated. To further account for this discrepancy, we took an additional step and included in our dataset the published fungal BGCs that have been linked to known compounds but are not yet incorporated into MIBiG. For instance, olivetoric acid in *Pseudevernia furfuracea* [40] and gyrophoric acid in *Umbilicaria* species have both recently been linked to specific BGCs through bioinformatic and phylogenetic approaches. We used these as references and excluded BGCs clustering with them from the list of potentially novel BGCs.

Clustering with the MIBiG reference BGC indicated that it potentially encoded a similar compound. We identified the usnic acid, 6-methylsalicylic acid, 6-hydroxymellein and orcinol GCFs based on clustering with MIBiG reference BGCs [54] (Figs 3a and 4). In addition, we identified atranorin [11], a tridepside GCF [55] and olivetoric/physodic acid [41] GCF (Material S3A and S3B). These BGCs have been characterized from lichens based on experimental and/or phylogenetic evidence. The phylogenetic grouping of *P. furfuracea* and *Umbilicaria* spp. was used as a reference for this characterization.

 BGCs that do not cluster with a MIBiG reference BGC or a pre-characterized lichen BGC are potentially novel (including those that may be active but have not yet been linked to a known metabolite).

### Metabolite profiling and molecular networking

All the lichen specimens in the current study (Material S1) were carefully identified by lichen taxonomists (using both morphology and DNA barcoding) and deposited in recognized herbaria. To identify potential depsides and depsidones in the lichen extracts, and to infer the taxonomic depth of the metabolites, we conducted MS analysis in negative ion on 109 samples, representing 80 species, of which genomic data were available for 27 LFF. We performed integrative omics analysis to identify the putative BGC responsible for the synthesis of the identified depsides or depsidones.

The lichen extracts were systematically surveyed for metabolites to perform large-scale MS profile comparisons among *Lecanoromycetes* families and infer the relation between the degree of taxonomic diversity and secondary metabolite diversity (Material S6).

For each specimen, lichen thallus materials (ca. 15 mg) were ground into powders under liquid nitrogen. Metabolites were extracted three times from ground powders with acetone (800 µl each time), and then, the extracts were combined and evaporated. Dried residues were reconstituted in 2 ml solvent mixture of methanol and acetonitrile (50:50, v/v), and a 50 µl aliquot was diluted 20 times with the same solvent mixture and filtered (0.2 µm, PTFE) before liquid chromatography-MS (LC-MS) analyses.

LC-MS measurements were carried out on a Waters Acquity ultra-high performance liquid chromatography system coupled to a SYNAPT XS quadrupole time-of-flight high-resolution mass spectrometer with an electrospray ionization interface. Chromatographic separation of lichen specialized metabolites was performed on a Kinetex EVO C18 column (150×2.1 mm, 1.7 µm). The mobile phase consisted of 0.1% formic acid in water (solvent A) and 0.1% formic acid in acetonitrile (solvent B). A gradient elution was used as follows: 0–0.5 min, 10%B; 0.5–10 min, linear gradient from 10%B to 100%B; 10–11 min, 100%B; 11–11.1 min, linear gradient from 100 to 10 %B; 11.1–13 min, 10%B. Flow rate was 0.45 ml min^−1^, and 5 µl test solution was injected. MS data on lichen acids were acquired from the negative ion mode (mass range 100–1200 m/z). Raw MS data were acquired in continuum mode and converted to centroid data using the accurate mass measure function embedded in the software MassLynx v4.2. Lock mass for negative ion modes was 554.2615 m/z, respectively. Centroid data were further converted to the mzML format using MSconvert [56].

The mzML files were exported and uploaded to Global Natural Product Social Molecular Networking (GNPS) [57] for classic molecular networking, with each group separated by family. The tolerance of both precursor ion mass and fragment ion mass was 0.02. The molecular networks were visualized using Cytoscape 3.9.1. The molecular network (Fig. 4) was constructed from metabolomics data generated in negative ion mode, covering a broad spectrum of LFF-unique substances, e.g. depsidones, depsides, dibenzofurans, pulvinic acid derivatives and aliphatic lactones. Compounds sharing similar mass spectra are clustered, and major chemical groups are visualized. Each node represents a lichen metabolite, and colours in the node indicate its presence in lichen-forming fungal families. Lichen metabolites were initially annotated by comparing our in-house dataset with reference data deposited in GNPS and further dereplicated by literature search.

## Results

### High diversity of BGCs in lichen-forming fungal genomes

AntiSMASH detected 5542 BGCs in 111 LFF (Table S1). The number of BGCs varied at the family and genus levels, with *Sarrameanaceae* being the family with the fewest BGCs (14 BGCs) and *Pyrenulaceae*, *Physciaceae* and *Trypetheliaceae* being the most BGC-rich families (~70 BGCs per taxon) (Fig. 1a and b, Material S2). The greatest number of BGCs (95 BGCs) was detected in *Canoparmelia texana* (*Parmeliaceae*) and the lowest number (14 BGCs) in *Caeruleum heppii* (*Acarosporaceae*) (Fig. 1a).

Lichenized fungi contain an average of 47±20 BGCs per genome, ~23±11 PKSs and 15±7 NRPSs/taxon (Fig. 1b), making the proportion of PKS to NRPS clusters 3 :2 (2,622:1,726), respectively. The most dominant BGC class in lichenized fungi is PKSs, accounting for ~50% of the total BGCs, followed by NRPSs (~23%), RiPPs (16%) and terpenes (~12%) (Material S2). We surveyed the complete set of BGCs in LFF genomes and found that the metabolic potential varied among families, with *Physciaceae* constituting the richest source of terpenes and NRPSs, whereas *Pyrenulaceae* and *Trypetheliaceae* were the richest sources of PKSs (Fig. 1b, Material S2).

### Potentially novel BGCs

**Fig. 2.**
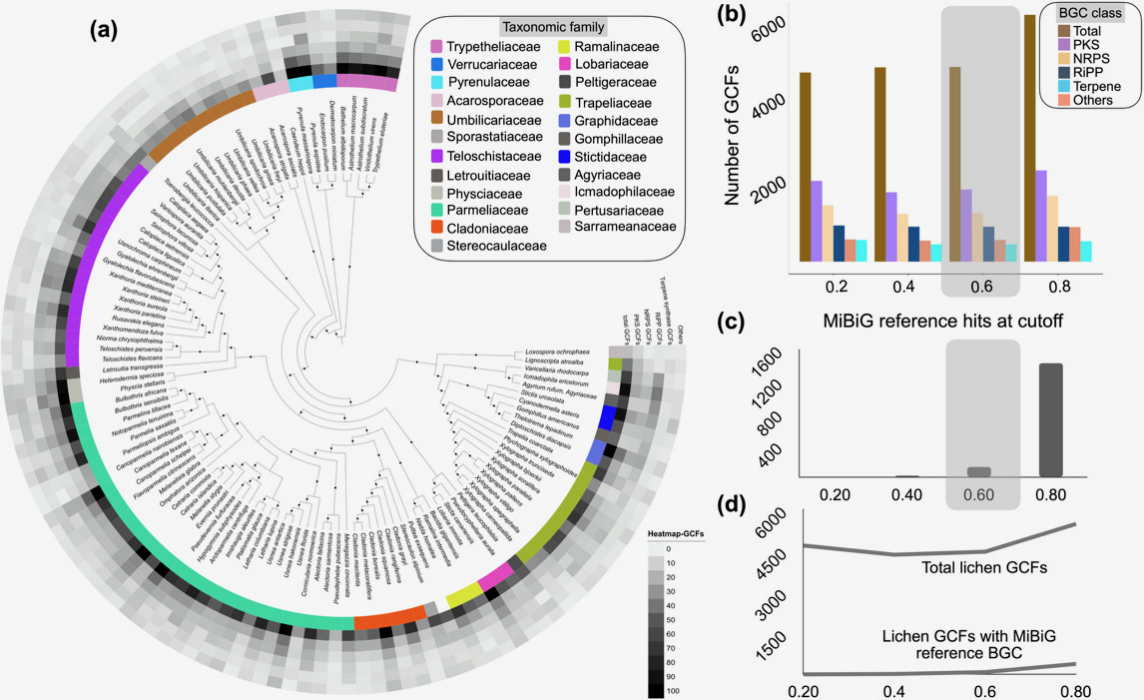
Novel biosynthetic genes encoded in genomes of selected LFF. (**a**) ML phylogenomic tree highlighting the number of unique GCFs identified for each BGC class in LFF. The inner coloured ring corresponds to the taxonomic family within *Lecanoromycetes*. The heatmaps represent the number of GCFs (total, PKS, NRPS, terpene, RiPP and other BGCs including indoles, PKS-NRPS hybrids and isocyanide synthase, from inside to outward). (**b**) Bar plots showing the total number of GCFs retrieved and those with similar clusters found in the MIBiG database (**c**), using varying thresholds in the BiG-SCAPE analysis, categorized by BGC classes. (**d**) Gap between the total LFF GCFs and those associated with characterized BGCs in the MIBiG database.

BiG-SCAPE generated the network for each BGC class – within each network, similar BGCs were organized into GCFs, while unique BGCs remained as singletons. LFF have a vast repertoire of GCFs which comprise only a few BGCs (Fig. 2a and b, Material S3A and B). Most of the lichen GCFs are unique and are not similar in sequence or cluster composition to previously known BGCs present in standard curated databases (antiSMASH [58] and MIBiG [36]) (Fig. 2c and d, Material S3A).

Among the BGC classes, only the PKS BGCs showed similarity to BGCs in MIBiG, while all other classes, including NRPS, did not. This could be because polyketides are among the most prevalent as well as the most studied molecular classes in LFF [12,24, 27, 59]. Of ~1,500 PKS GCFs, only 3 clustered with BGCs listed in MIBiG (at a BiG-SCAPE clustering threshold of 0.6, Fig. 2b), namely 6-methysalicyclic acid, 6-hydroxymellein and usnic acid (Material S4). The network approach facilitated the identification of these pathways/BGCs in many LFFs for the first time.

### RiPPs

We identified 1,186 RiPP BGCs in our dataset, clustering into 987 GCFs (Material S3A and S3B). These RiPP BGCs showed low similarity (8–30 %) to those known from *Lecanoromycetes* or other fungi (asperipin, ustiloxin and phomopsin BGCs), suggesting that LFF RiPP derivatives might be structurally and functionally more diverse than those of non-lichenized fungi. Like PKSs, the diversity of RiPP BGCs varies within *Lecanoromycetes*, with RiPP being the predominant BGC class in *Agyriaceae*, *Icmadophilaceae*, *Pertusariaceae* and *Stictidaceae*, outnumbering PKSs and NRPSs.

### Deorphanizing BGCs and metabolites: clustering-based linking of genes to molecules

**Fig. 3.**
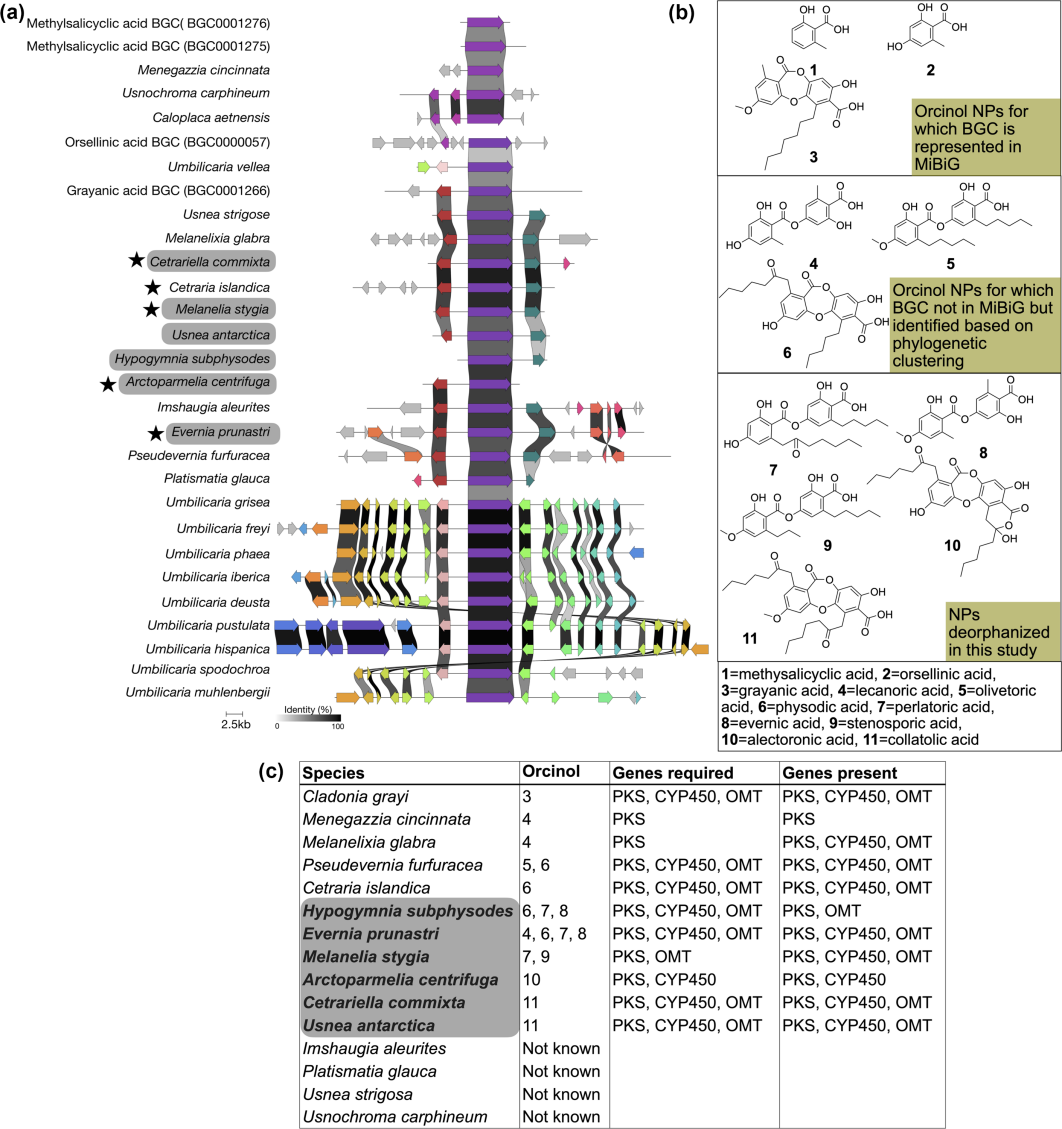
(a) Clinker plot showing the symmetry and synteny between three orcinol GCFs (6-methylsalicylic acid (1), grayanic acid (3) and olivetoric acid (5) GCF) and (b) the compounds produced by these taxa. Taxa highlighted with grey boxes represent those from which the natural products were deorphanized, while taxa marked with stars indicate those analysed using MS. The orcinol NPs are among the most structurally and functionally versatile compounds of LFF. Here, we identified the putative BGCs for five compounds. Notably, each taxon contains only one putative depside/depsidone gene cluster, although the organism may produce one or more structurally related compounds. (**c**) The compounds produced by some of the common LFF and the genes required to produce the compound of this class. The taxa in bold mark the species from which the compounds were deorphanized.

Most known bioactive compounds of LFF belong to the orcinol or orcinol derivatives class [18,60,62]. However, the corresponding BGCs are known only for a few compounds [21]. Based on the GCF recovered in the PKS network (Fig. 3a), we for the first time infer most-likely BGCs for the following five orcinol metabolites: perlatolic acid (compound 7, Fig. 3b), evernic acid (compound 8, Fig. 3b), stenosporic acid (compound 9, Fig. 3b), alectoronic acid (compound 10, Fig. 3b), collatolic acid (compound 11, Fig. 3b) and their derivatives (Fig. 3b). This inference is based on correlative omics, integrating species-specific metabolite production patterns with clustering of BGCs with pre-characterized orcinol derivative gene clusters, including 6-methylsalicylic acid (compound 1, Fig. 3b), grayanic acid (compound 3, Fig. 3b) and olivetoric acid (compound 5, Fig. 3b). Notably, all these NPs have unique chemical properties despite sharing a similar core structure (Fig. 3b) [61,63]. Of these, grayanic acid and 6-methylsalicylic acid clusters are present in MIBiG, whereas the olivetoric acid BGC was recently identified based on *in silico* predictions [41]. The clusters from different taxa grouped with grayanic acid/olivetoric acid BGCs that encode the different orcinol compounds (Fig. 3c). Since we retrieved one candidate BGC per taxon, and only one main metabolic product within this compound class has been reported for these taxa, we are certain that the BGC codes for the depside/depsidone reported from the lichen.

In addition, we identified putative BGCs for two PKS-derived bioactive NPs, usnic acid and atranorin, from several taxa. The BGCs provided here differ in sequence conservation as well as gene composition from previously reported usnic acid and atranorin BGCs [11,22, 28, 41]. The taxa and BGCs reported in this study represent novel sources of these metabolites. Given the diversity in BGC and gene sequences, they may encode slightly different structural and functional variants of these compounds. The GenBank files of these clusters are available as Material S5.

### Deorphanizing BGCs and metabolites: metabolite profiling and correlative metabolomics

**Fig. 4.**
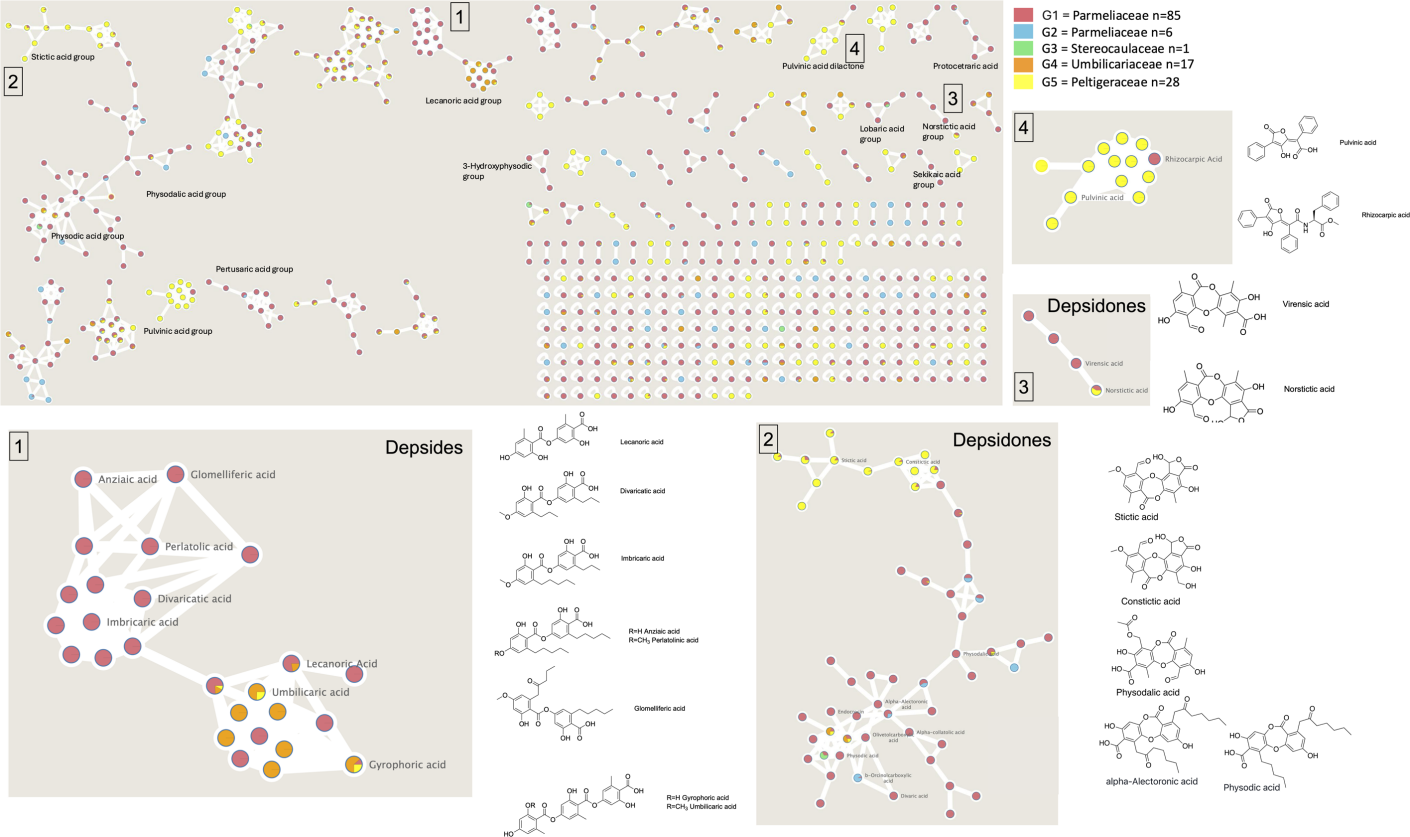
Classical molecular networking based on LFF extracts. Different colours represent taxonomic families within the *Lecanoromycetes*. Network 1 consists of depsides from the *Parmeliaceae*, *Umbilicariaceae* and *Peltigeraceae* families. Network 2 contains depsidones. Network 3 comprises depsidones from the families *Parmeliaceae* and *Umbilicariaceae*. Network 4 includes molecules with structures similar to pulvinic acid, found in the *Peltigeraceae*, and rhizocarpic acid, found in the *Parmeliaceae*. Orcinol compoiund numbers in 2c refer to those shown in Fig. 2b.

We performed integrative omics analysis to identify the putative BGCs responsible for the synthesis of the identified depsides or depsidones. Using PKS clustering and BGC-to-compound structure correlations, we linked MS spectra to putative BGCs for five bioactive compounds. Specifically, we deorphanized the following NPs: alectoronic acid (compound 10, Fig. 3b), collatolic acid (compound 11, Fig. 3b), evernic acid (compound 8, Fig. 3b), stenosporic acid (compound 9, Fig. 3b) and perlatolic acid (compound 7, Fig. 3b). This was done by employing a multifaceted approach based on the BGC class and compound structure match, gene clustering and gene cluster similarity. For each compound, we systematically narrowed down the candidates to a single, most likely BGC. This congruence between the molecule structure, gene cluster and phylogenetic clustering provides strong evidence of identified BGCs being the exclusive candidates responsible for the biosynthesis of the respective compounds. Molecular network (Fig. 4) was constructed from metabolomics data. Compounds with similar mass spectra were clustered, and major chemical groups were visualized.

### Taxonomic breadth of four common, bioactive LFF metabolites

We profiled a total of 80 species (109 samples) to analyse their metabolites and identify the potential depsides and depsidones secreted by them. In the studied species, we found that stictic acid, a *β*-orcinol depsidone, is the predominant compound secreted by the members of the family *Peltigeraceae*. Notably, it is not exclusive to this family but also produced by certain members of *Parmeliaceae*, e.g. *Usnea* spp. and *Acarospora* spp. [64,65]. In contrast, members of studied *Parmeliaceae* taxa secrete a diverse array of orcinol didepsides and didepsidones, as lecanoric acid (compound 4, Fig. 3b), olivetoric acid (compound 5, Fig. 3b), physodic acid (compound 6, Fig. 3b) and others. *Umbilicariaceae* lichens, on the other hand, primarily produce orcinol tridepsides as gyrophoric acid and derivatives. This suggests that distantly related families may harbour structurally and functionally diverse metabolites and that distantly related genera represent untapped sources of unique NPs. Similarly, closely related taxa and genera are potential sources of known compounds or variants of these compounds. The taxonomic distribution of these bioactive compounds reveals a strong pattern between specific taxonomic families and their associated metabolites.

## Discussion

This study provides the first systematic comparison of BGC diversity within LFF and explores their metabolic uniqueness. We analysed LFF genomes to bioinformatically characterize their metabolic diversity, grouping them by the chemical families of encoded compounds and identifying novel/unique biosynthetic gene clusters. Interestingly, we found RiPPs, previously known from bacteria and *Basidiomycetes* fungi, to constitute about 15–20% of lichen biosynthetic space. This is the first study reporting the RiPP BGC contribution to total biosynthetic gene space of lichens. Concurrently, we performed correlative metabolomics on five metabolites to establish gene-to-molecule links and inferred the taxonomic breadth of widespread bioactive lichen metabolites, such as stictic acid and lecanoric acid.

### High diversity of BGCs and GCFs in lichen genomes

We found that on average, LFF contain ~47±20 BGCs, as per literature but the number of identified compounds per species is usually fewer than 10 [21,59, 61, 66], indicating a majority of clusters are either temporally silent or orphan, or the BGC is active but the metabolite goes undetected by regular detection techniques as TLC/HPLC [21,22]. Even though most of these BGCs are still orphan, their presence in high number in LFF is likely an evolutionary strategy rather than the accumulation of silent and non-functional BGCs. A recent study showed that specialization in metabolism is the primary feature of the genetic turnover in the evolution of fungi [67]. Interestingly, while Metazoa, on average, accumulated genes from diverse functional categories, in fungi only a few categories showed net gains during evolution, one of which is secondary metabolism [67]. The bioinformatic identification of an organism’s entire BGC landscape provides opportunities for the discovery of novel structural diversity that can lead to promising leads.

 While the majority of BGCs are predicted to encode PKSs, which is consistent with the known diversity of orcinol derivatives in LFF, we discovered many unique GCFs for BGC classes RiPPs, NRPSs and terpenes (Fig. 2a, b). For instance, although the majority of reported NPs from LFF are PKS-derived (melanins, usnic acid, grayanic acid, olivetoric acid, gyrophoric acid, umbilicaric acid etc.) [25,68,70], a typical LFF BGC landscape is biosynthetically diverse, encompassing three to five classes of BGCs [27,28, 71, 72] (Fig. 1a and b). This further highlights that only a small fraction of the chemical diversity in LFF has been explored to date. Moreover, research on lichen biosynthesis has focused on PKSs, but the diversity of other classes, with the exception of terpene BGCs [38], remains largely unexplored. The BGCs and the BGC families reported here are valuable resources for elucidating thus far neglected classes of biosynthetic compounds.

### RiPPs

A notable outcome of our study is the significant representation of RiPP-BGCs in LFF genomes, comprising 16% of the identified BGCs. Although RiPP-BGCs account for a substantial portion of the bacterial biosynthetic landscape, approximately one-fourth of all BGCs and ranking as the second most predominant class after NRPSs, their representation in fungal genomes was estimated to be markedly lower, at ~1%, based on data from 2,000 to 5,000 species [7,73, 74]. This may be attributed to the limitations of detection algorithms, coupled with the limited understanding of fungal RiPP biosynthesis [74]. For example, while plant and bacterial RiPPs have been extensively studied, the first fungal RiPP was not discovered until 2007, and only a handful have been characterized since then [74]. Our study is the first to show that the RiPP BGCs contribute more to the total LFF BGCs than previously recognized.

### Biosynthetic diversity of LFF compared to bacteria and non-lichenized fungi

We found that LFF fungi have ~50 BGCs and about 40 GCFs per species. In contrast, non-lichenized fungi were shown to have around 23 BGCs and 0.5 GCFs (123,939 BGCs and 2935 GCFs detected in 5,588 fungal genomes [73], . As a single lichen, BGC can encode more than one compound by selectively activating different combinations of genes within the cluster [21,55]; the total biosynthetic potential of lichens may surpass the number of biosynthetic gene clusters present in their genomes. For example, in *Umbilicaria*, the same BGC can produce structurally and functionally distinct compounds, such as the core PKS-derived product, its methylated form and an oxidized variant [34]. Furthermore, given the data, it seems that lichenized fungi are ~10 times richer and more diverse in their biosynthetic capacity than bacteria, which on average contain 5.4 BGCs and 3.5 GCFs per taxon (1,185,995 BGCs present in 217,647 species [37]). This is particularly interesting because bacteria currently constitute the most prominent source of drugs. Considering the pressing demand for novel drugs, lichenized fungi constitute a very promising reservoir to explore.

 Previous studies show that the predominant BGC class in fungi is NRPS (~42% of BGCs are NRPS clusters) [38]. Interestingly, we found that in LFF, PKSs are the predominant BGC type (~38 %), followed by NRPS (23%) (Fig. 1c). The chemical repertoire varied among families, with *Physciaceae* constituting the source of most novel terpenes and NRPSs and *Pyrenulaceae* and *Trypetheliaceae* being the richest sources of PKSs (Fig. 1), highlighting the complementarity of the BGC catalogue of lichenized fungi.

### Deorphanized natural products

Only a few PKS GCFs recovered in our network analysis grouped with a characterized BGC, for instance, 6-hydroxymellein and usnic acid (Material S4). We present novel sources, including BGCs and taxa, for these compounds.

 6-Hydromellein displays broad-spectrum antimicrobial activity against both bacteria and fungi [23]. Usnic acid, on the other hand, is one of the most studied lichen metabolites and displays anti-inflammatory, analgesic, healing, antioxidant, antimicrobial, antiviral and anti-UV properties [65,75]. The 6-hydroxymellein and usnic acid clusters from different organisms presented here (Material S4) provide a premise for combinatorial mellein biosynthesis to adapt the product for medicinal use.

We found that, apart from PKS, the most common genes present in the LFF BGCs are CYP450 and oxidase. These genes are potentially involved in the modification of the compound synthesized by PKS to produce the final compound [11,24], adding to the chemical diversity of the organism, e.g. chemosyndrome in *P. furfuracea* and PKS codes for olivetoric acid, which, when oxidized by CYP450, produces the corresponding depsidone physodic acid (compound 6, Fig. 3b) [41].

 In most cases, the compound structure aligns with the expected biosynthetic gene content (Fig. 3b). When multiple compounds are produced, different genes contribute to the synthesis of specific compounds. For example, *Evernia prunastri* produces lecanoric acid (compound 4, Fig. 3b), physodic acid (compound 6, Fig. 3b), perlatolic acid (compound 7, Fig. 3b) and evernic acid (compound 8, Fig. 3b). The synthesis of lecanoric acid requires only PKS, while physodic acid synthesis involves both PKS and CYP450, and evernic acid synthesis depends on PKS and OMT.

We also found that tailoring enzymes in the orcinol clusters are omnipresent, although the NP produced may not require these enzymes. Lichens with orcinol BGC likely produce additional NP variants beyond those that have been described based on TLC and HPLC. A recent study implementing MS on lichens reported novel NP variants of the compounds in *Hypogymnia subphysodes*, *E. prunastri* and *Ophioparma ventosa* [43].

### Unique BGCs: potential sources of novel natural products

Our BGC exploration and comparison suggested that 98% of the lichen GCFs are exclusive (Material S3B). Additionally, each LFF contained several unique GCFs not found in other lichenized fungi (Material S3A, S3B). However, the most diverse groups of metabolites are predicted to be produced by the taxa belonging to *Parmeliaceae*. Some families are particularly rich in unique GCFs, making them the most promising sources of novel biosynthetic diversity. We propose that the orphan clusters are the most interesting targets for drug discovery efforts.

### Correlative metabolomics

In this study, we perform the metabolite survey of 80 taxa (109 samples) using MS (Material S6). We found both known and previously unidentified metabolites in the lichen MS profiles and performed large-scale comparison of MS profiles in relation to the taxonomy.

We find a correlation between taxonomic distance and the production of distinct secondary metabolite families. Specifically, in the studied species, out of four networks, stictic acid, a *β*-orcinol depsidone, is predominantly produced by the members of the family *Peltigeraceae*. Instead, members of *Parmeliaceae* secrete a great variety of orcinol didepsides and didepsidones, as lecanoric acid, physodic acid, olivetoric acid etc. *Umbilicariaceae* lichens, on the other hand, primarily produce orcinol tridepsides as gyrophoric acid and derivatives. The taxonomic range of these bioactive compounds revealed a strong pattern between specific taxonomic families and their associated compounds. We advocate that chances of discovering novel metabolites are greater in distantly related taxa.

## Conclusions

Our study reveals that lichen-forming fungi are a rich source of novel natural products, with ~98% of their BGCs being potentially novel/uncharacterized to date. By exploring the biosynthetic landscape of lichens, we found that PKS BGCs are predominant in lichens, whereas non-lichenized fungal BGCs are mainly composed of NRPS. Furthermore, we demonstrate for the first time that ribosomal peptide-related BGCs constitute about 20% of LFF BGCs. Our study categorizes lichen BGCs into known, unknown but widespread, and novel (yet uncharacterized) groups. We propose that the comparative omics and genome mining approach employed in our study provides a foundation for advancing biosynthetic research in non-model organisms and fosters further exploration of microbial dark matter.

## Supplementary material

10.1099/mgen.0.001569 Supplementary Material 1.

10.1099/mgen.0.001569 Supplementary Material 2.

## Abbreviations

BGCs: biosynthetic gene clusters
BiG-SCAPE: Biosynthetic Genes Similarity Clustering and Prospecting Engine
GCFs: gene cluster families
LC-MS: liquid chromatography-MS
LFF: lichen-forming fungi
ML: maximum likelihood
NPs: natural products
NRPSs: non-ribosomal peptide synthetases
PKSs: polyketide synthases
RiPPs: ribosomally synthesized and post-translationally modified peptides
