# Supplementary material for: Paired-omics-based exploration and characterization of biosynthetic diversity in lichenized fungi
Source: Microb Genom. 2025 Dec 17;11(12):001569. doi: 10.1099/mgen.0.001569 (PMC12711211; doi:10.1099/mgen.0.001569)

Supplementary material S3 A) Number of Gene cluster families for all the BGC classes per taxa identified by the BiG-SCAPE; B) Number of Gene cluster families per taxa identified by the BiG-SCAPE to demonstrate the sources of most unique BGCs in lichens

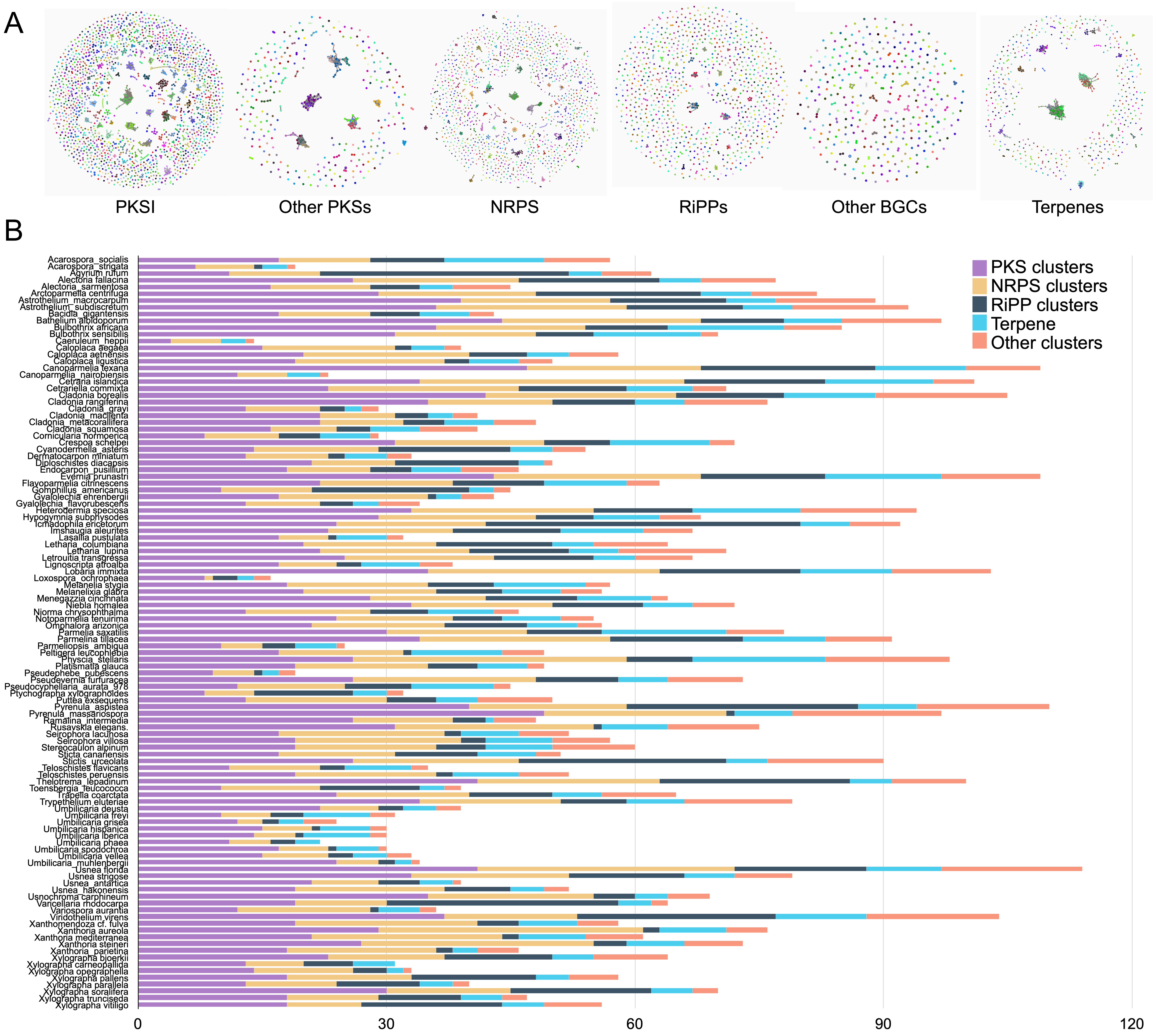

Supplementary material S4. BGCs identified based on clustering with atranorin, usnic acid and gyrophoric acid gene clusters.

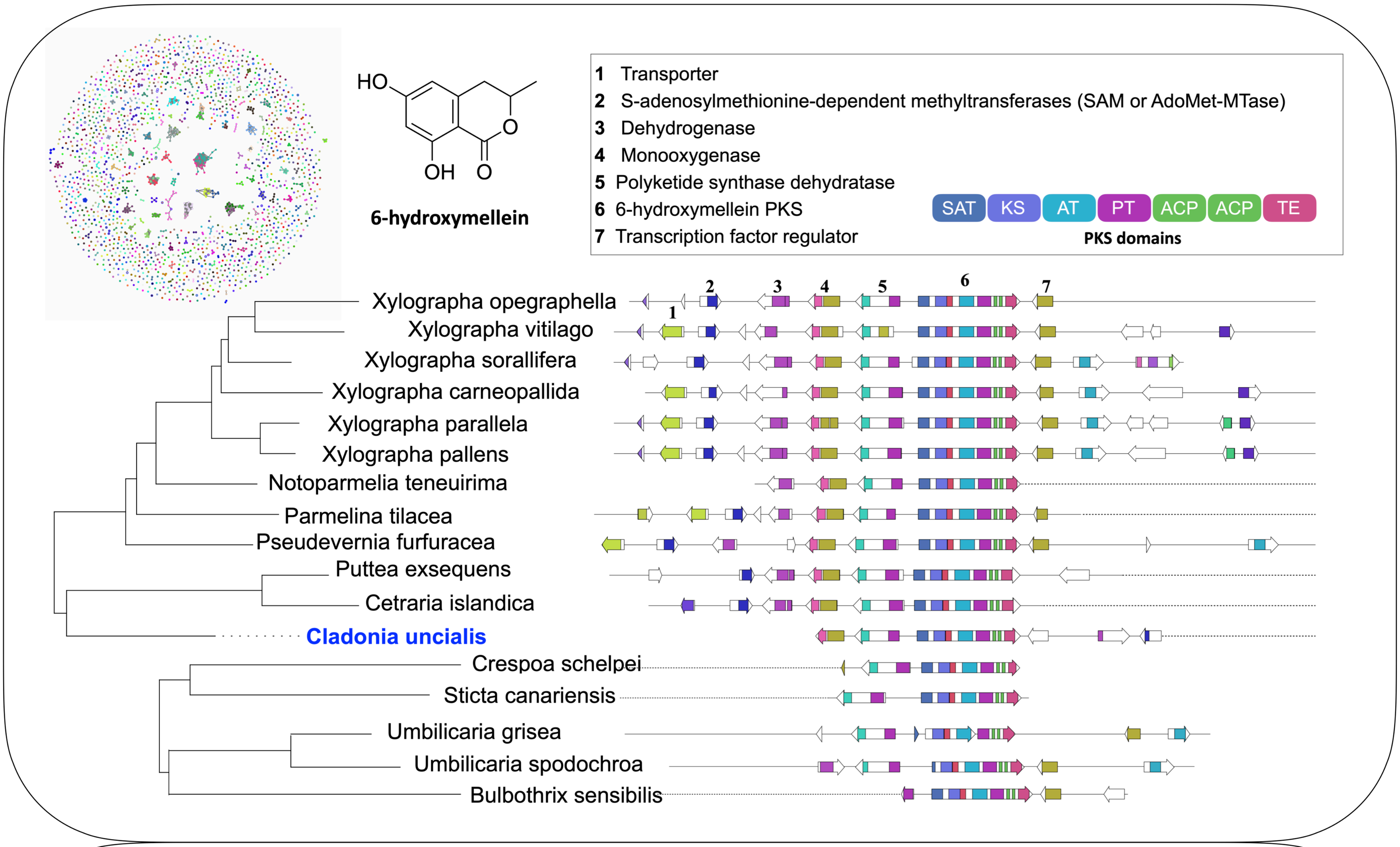

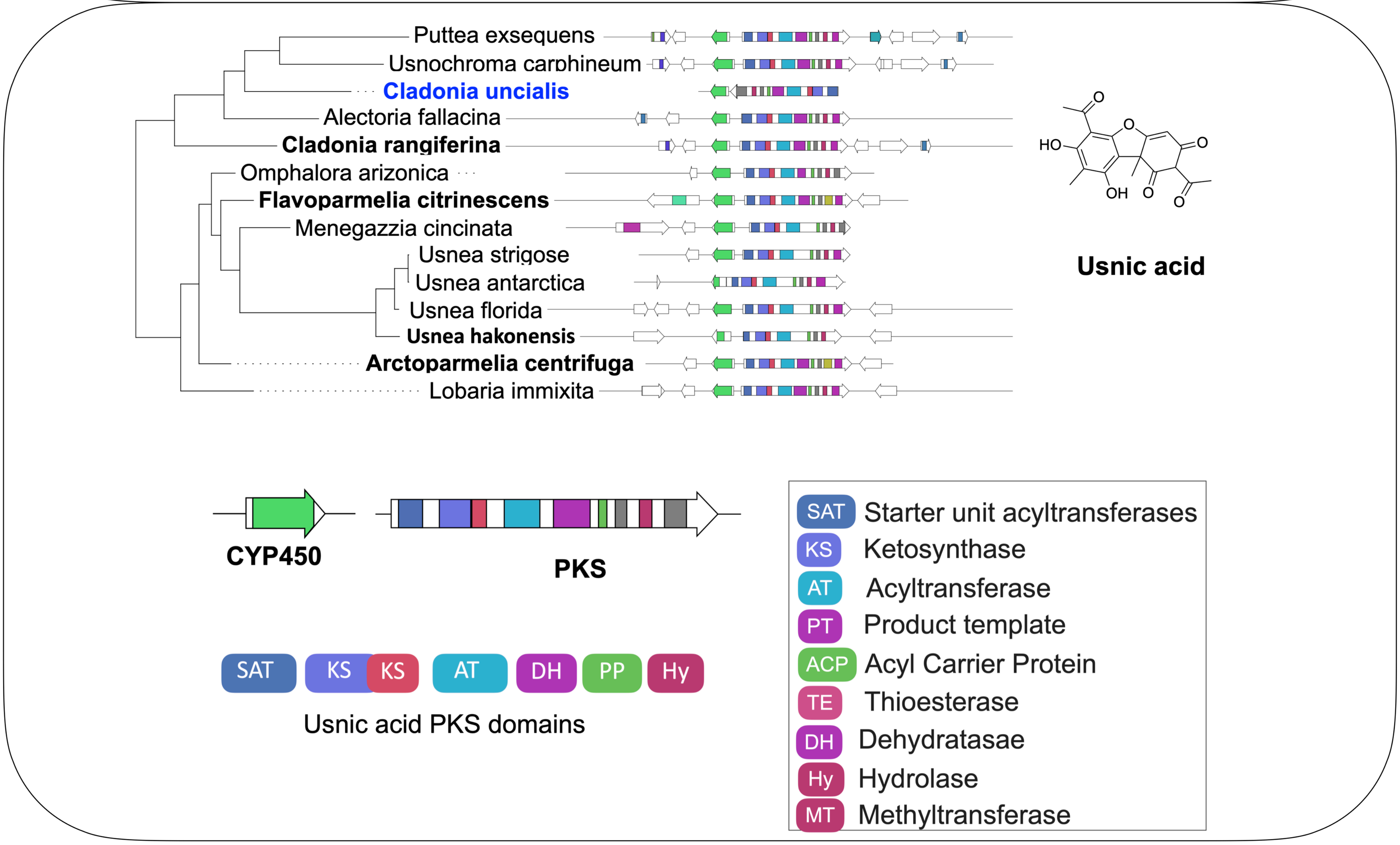

Supplement: Supplementary Material 1. [file mgen-11-01569-s001.pdf]
